# Short-term survivors in glioblastomas with oligodendroglioma component: a clinical study of 186 Chinese patients from a single institution

**DOI:** 10.1007/s11060-013-1311-3

**Published:** 2013-11-22

**Authors:** Haihui Jiang, Xiaohui Ren, Junmei Wang, Zhe Zhang, Wenqing Jia, Song Lin

**Affiliations:** 1 Neurosurgery, Beijing Tiantan Hospital, Capital Medical University, Beijing, 100050 China; 2 Pathology, Beijing Neurosurgical Institute, Capital Medical University, Beijing, 100050 China

**Keywords:** GBMO, Short-term survivors, Polysomy

## Abstract

This study was designed to display the molecular genetic features of short-term survivors in glioblastomas with oligodendroglioma component (GBMO). A total of 186 patients with histological diagnosis of primary gliomas, including 11 GBMO-STS (short-term survivors, survival ≤12 months), 29 GBMO-LTS (relatively long-term survivors, survival >12 months), 36 anaplastic oligoastrocytoma (AOA) and 110 glioblastoma multiforme (GBM), enrolled in the study. An evaluation form was developed and used to document molecular pathological, clinical and treatment-associated parameters between subgroups. Kaplan–Meier plots for survival showed that the median progression-free survival (PFS) and overall survival (OS) of GBMO-STS were 5.0 and 10.0 months, respectively. Intergroup comparison revealed that the GBMO-STS harbored the most dismal prognosis than those with AOA, GBMO-LTS or GBM (*P* < 0.001 for PFS, *P* < 0.001 for OS, respectively). Cox regression analyses revealed that 1p/19q co-deletion and 19p polysomy were independent prognostic factors (*P* < 0.05). Pearson’s Chi square test demonstrated GBMO-STS exhibited lower 1p/19q co-deletion, IDH1 mutation rates than AOA or GBMO-LTS (*P* = 0.032, *P* = 0.045 for 1p/19q co-deletion; *P* = 0.034, *P* = 0.005 for IDH1 mutation, respectively) but higher chromosome 1q, 19p polysomy rates compared with AOA or GBM (*P* = 0.037, *P* = 0.030 for 1q polysomy; *P* = 0.017, *P* = 0.011 for 19p polysomy, respectively). Patients with glioblastomas with oligodendroglioma component concurrent with polysomy for chromosomes 1 and 19 always confers an unfavorable prognosis which needs our extra attention in clinic.

**Electronic supplementary material:**

The online version of this article (doi:10.1007/s11060-013-1311-3) contains supplementary material, which is available to authorized users.

## Introduction

The latest WHO classification of tumors of central nervous system (CNS) introduced an entity–AOA with necrosis was to be diagnosed as glioblastomas with oligodendroglioma component (GBMO). As a new pathology entity, the studies devoted to revealing the prognosis of GBMO were relatively limited.

Increasing evidence suggested GBMO was a heterogeneous group with considerable survival variant. Several reports found that the survival of GBMO was significantly longer than glioblastoma multiforme (GBM) whose median overall survival (OS) was only 12–15 months in spite of multimodal aggressive treatment, comprising surgical resection, local radiotherapy and systemic chemotherapy [1–3]. Nevertheless, reports showed GBMO without significant prognosis advantage over GBM were also consecutively published in the latest years [4–6]. In the present study, we found a small fraction of patients who were formerly diagnosed with AOA but displayed OS ≤12 months. However, after the pathology re-evaluation, all these patients were confirmed as AOA with necrosis which should be classified into GBMO according to the latest WHO classification of CNS tumors.

GBMO displayed OS ≤12.0 months is a relatively rare event which is worthy of further inquiry. It has not been firmly established which, if any, of the molecular genetic aberrations is important for the pathogenesis of GBMO-STS represent prognostic factors. The identification of molecular genetic markers that are associated with survival in patients with GBMO would be beneficial for its diagnostic and prognostic potential. In this regard, we report a retrospective analysis of 186 primary high-grade gliomas recruited in the Beijing Tiantan Hospital Neurosurgery Department. In addition to basic clinical data, we evaluate the clinical characteristics and screen for glioma associated genetic aberrations, i.e. 1p/19q co-deletion, IDH1 mutation, MGMT promoter methylation, PTEN, p53, Ki-67, EGFR, VEGF expression, as well as chromosome 1q, 19p polysomy.

## Materials and methods

### Patients

A total of 186 patients (male 111 and female 75) with histological diagnosis of primary supratentorial high-grade gliomas (including 36 AOA, 11 GBMO-STS, 29 GBMO-LTS and 110 GBM) in Beijing Tiantan Hospital from May 2008 to May 2011 were enrolled in the study. The secondary GBM were excluded. All patients provided written informed consent for the current study and the clinical study was approved by the Medical Ethics Committee of Capital Medical University. The mean age of this cohort was 47.5 ± 12.5 years at the time of surgery. All specimens were independently re-evaluated by three experienced neuro-pathologists according to the 2007 WHO classification of the CNS tumors [7]. In case of a discrepancy, the three observers simultaneously reviewed the slides to achieve a consensus. Patients who underwent needle biopsies prior resection, and/or prior adjuvant therapy (radiotherapy or chemotherapy) were excluded. These were done to create a more uniform patient population which could be propitious to the study.

### Treatment

All the patients with gliomas, in our institution, were treated according to the National Comprehensive Cancer Network (NCCN) guideline. Patients, in our department, once pathologically diagnosed with high grade glioma, systemic chemotherapy and radiotherapy will be attempted after operation. Maximal tumor bulk resection while preserving the vital eloquent cortex was the principle goal during operation. Intraoperative subcortical electrical stimulation was performed when necessary. Extent of resection was assessed by the intraoperative ultrasound. Postoperative radiotherapy was routinely delivered to patients within 1 month after operation. The total dose was 60 Gy, which was divided into 30 daily fractions of 2 Gy each. Meanwhile, postoperative chemotherapy was given; the common course of chemotherapy was 4–6 cycles which depended on the tolerance of toxic effect. The adjuvant chemotherapy drugs were mainly nimustine (ACNU) or temozolomide (TMZ).

### Recorded variables

The clinical, operative, and hospital course records of 186 patients who met the inclusion criteria were retrospectively reviewed. The following information was recorded including patient’s age, gender, removal degree, location of tumor, adjuvant therapy, and molecular parameters. The molecular parameters in this study included 1p/19q, IDH1, MGMT, PTEN, p53, Ki-67, EGFR, and VEGF. The status of chromosomes 1 and 19 was detected by fluorescence in situ hybridization (FISH) method, and IDH1 was sequenced. PTEN, p53, Ki-67, EGFR, VEGF expression were detected by immunohistochemical method. The MGMT promoter methylation was analyzed by methylation-specific PCR (MSP).

### Assessment of 1p/19q status by the fluorescence in situ hybridization (FISH) method

1p/19q co-deletion was detected by FISH method as described previously [8]. Tumors with more than 30 % of nuclei showing DNA loss were defined as tumor with chromosomal loss. The tumor was considered to have polysomy if >30 % of nuclei showed more than two 1q and 19p signals.

### IDH1 sequence analysis

Genomic DNA was isolated from snap-frozen tissue using the QIAmp DNA mini-kit, as described by the manufacturer (Qiagen). A fragment of 254 bp length spanning the catalytic domain of IDH1 including codon 132 was amplified using the sense primer IDH1 F: 5′-ACCAAATGGCACCATACG-3′ and the antisense primer IDH1 R: 5′-TTCATACCTTGCTTAATGGGG-3′. PCR using standard buffer conditions, 30 ng of DNA and GoTaq DNA Polymerase (TaKaRa, Japan) employed 35 cycles with denaturing at 95 °C for 30 s, annealing at 54 °C for 45 s and extension at 72 °C for 50 s in a total volume of 25 μL. The PCR amplification product was sent to Beijing Tianyi Huiyuan Bioscience and Technology Incorporation for sequencing.

### Immunohistochemical analysis

Evaluation of PTEN, p53, Ki-67, EGFR, VEGF was detected by immunohistochemistry (IHC) as described previously [9]. The expression levels were based on the percentage of immunopositive cells (negative <10 % of tumor cells; positive ≥10 % of tumor cells) (Table S1).

### MGMT promoter methylation analysis

Genomic DNA was isolated from frozen tumor tissue by using Qiagen kit (Qiagen, Valencia, CA). MGMT promoter methylation was analyzed by MSP. Tumor DNA (2 μg) was treated with sodium bisulfite using the CpG genome DNA modification kit (Qiagen). The primer sequences for the unmethylated reaction were 5′-TTTGTGTTTTGATGTTTGTAGGTTTTTGT-3′ (forward) and 5′-AACTCCACACTCTTCCAAAAACAAAACA-3′ (reverse). For the methylated reaction, they were 5′-TTTCGACGTTCGTAGGTTTTCGC-3′ (forward) and 5′-GCACTCTTCCGAAAACGAAACG-3′ (reverse). The annealing temperature was 59 °C. The PCR products were separated on 4 % agarose gels. The investigators who selected and analyzed the glioblasoma samples were blinded to all clinical information. Pyrosequencing analysis was carried out by Gene Tech (Shanghai) Company Limited. The GBM samples [methylation values (10 %)] were considered as being methylated.

### Follow-up

The progression-free survival (PFS) was designated as the time period from the first operation to the time of tumor recurrence or evidence of progression based on magnetic resonance imaging (MRI). Patients who were recurrence-free at last follow-up were considered as a censored event in analysis. OS was defined as the period between the first operation and death or last follow-up. Patients who were still alive at last follow-up were considered as a censored event in analysis. All the survival data were collected mainly when patients visited the clinics and during the phone interview with patients and/or their relatives.

### Statistical analysis

SPSS 13.0 (SPSS for Windows, version 13.0 [SPSS Inc., Chicago, IL, USA]) was used for statistical analysis. Pearson’s Chi square test and Fisher’s exact test were used to compare the frequencies between groups. Kaplan–Meier method was used for survival analysis. Probability value was obtained from two-sided tests, with a statistical significance of *P* < 0.05.

## Results

### Basic characteristics

The basic clinical characteristics of these patients enrolled in the study were summarized in Table 1. A total of 186 patients with primary high-grade gliomas who were surgically treated in our institution met the inclusion criteria. There were 111 male and 75 female with a mean age of 47.5 ± 12.5 years old, including 36 (19.4 %) AOA, 11 (5.9 %) GBMO-STS, 29 (15.6 %) GBMO-LTS and 110 (59.1 %) GBM. The median follow-up period of the 186 patients was 13.5 months (range 1.0–42.0 months). A total number of 94 patients had dead.

**Table 1:** Clinical characteristics of AOA, GBMO-STS, GBMO-LTS, and GBM

Characteristic	Subgroup 1		*P* value	Subgroup 2		*P* value	Subgroup 3		*P* value
	GBMO-STS (*n* = 11)	AOA (*n* = 36)		GBM (*n* = 110)	GBMO-STS (*n* = 11)		GBMO-LTS (*n* = 29)	GBMO-STS (*n* = 11)	
Age (years)									
Median (range)	41.0 (14–58)	44.0 (30–68)	0.157	52.0 (12–70)	41.0 (14–58)	0.048*	43.0 (17–59)	41.0 (14–58)	0.348
Gender									
Male (%)	6 (54.5)	18 (50.0)		70 (63.6)	6 (54.5)		17 (58.6)	6 (54.5)	
Female (%)	5 (45.5)	18 (50.0)	0.792	40 (36.4)	5 (45.5)	0.789	12 (41.4)	5 (45.5)	1.0
Tumor location									
Temporal (%)	4 (36.4)	9 (25.0)		41 (37.3)	4 (36.4)		10 (34.5)	4 (36.4)	
Frontal (%)	4 (36.4)	15 (41.7)		37 (33.6)	4 (36.4)		11 (37.9)	4 (36.4)	
Parietal (%)	2 (18.1)	5 (13.9)		17 (15.5)	2 (18.1)		4 (13.8)	2 (18.1)	
Occipital (%)	0 (0.0)	1 (2.8)		4 (3.6)	0 (0.0)		0 (0.0)	0 (0.0)	
Insular (%)	1 (9.1)	3 (8.3)		6 (5.5)	1 (9.1)		4 (13.8)	1 (9.1)	
Others (%)	0 (0.0)	3 (8.3)	>0.05	5 (4.5)	0 (0.0)	>0.05	0 (0.0)	0 (0.0)	>0.05
Preoperative epilepsy									
Yes (%)	3 (27.3)	13 (36.1)	0.859	25 (22.7)	3 (27.3)	1.0	5 (20.7)	3 (27.3)	0.791
Preoperative KPS									
Median (range)	80 (60–90)	75 (50–100)	0.768	80 (50–100)	80 (60–90)	0.369	80 (60–100)	80 (60–90)	0.677
Tumor resection									
GTR (%)	6 (54.5)	25 (69.4)	0.583	52 (47.3)	6 (54.5)	0.645	18 (62.1)	6 (54.5)	0.942
Nimustine									
Yes (%)	11 (100.0)	34 (94.4)	1.0^#^	106 (96.4)	11 (100.0)	1.0^#^	26 (89.7)	11 (100.0)	0.548^#^
Radiotherapy									
Yes (%)	11 (100.0)	33 (91.7)	1.0^#^	102 (92.7)	11 (100.0)	1.0^#^	28 (96.6)	11 (100.0)	1.0^#^
Temozolomide									
Yes (%)	7 (63.6)	11 (30.6)	0.105	59 (53.6)	7 (63.6)	0.525	21 (72.4)	7 (63.6)	0.877

*KPS* Karnofsky performance score, *GTR* gross-total resection

^#^ Fisher’s exact test

* *P* < 0.05

### Survival analyses of AOA, GBMO-STS, GBMO-LTS and GBM

In the cohort 186 high-grade glioma, univariate analysis demonstrated that 1p/19q co-deletion, polysomy for 1q and 19p were associated with prognosis (*P* < 0.05) (Fig. S1). In multivariate Cox regression analysis, the presence of 1p/19q co-deletion and 19p polysomy were independent prognostic factors (*P* < 0.05) (Table 2). Unexpectedly, we haven’t found the prognostic value of MGMT promoter methylation, PTEN, p53, Ki-67, EGFR, VEGF expression (*P* > 0.05).

**Table 2:** Univariate and multivariate associations with survival for patients with high-grade gliomas

Parameter	Univariate analysis	*P* value	Multivariate analysis	*P* value
	Median survival (95 % CI) (months)		OR (95 % CI)	
Factors associated with PFS				
1p/19q co-deletion	N/A	<0.001	0.336 (0.176–0.643)	0.001
1q polysomy	9.0 (3.452–14.548)	0.003	–	
19p polysomy	7.0 (4.798–9.202)	<0.001	2.575 (1.608–4.124)	<0.001
Factors associated with OS				
1p/19q co-deletion	N/A	0.003	0.319 (0.134–0.760)	0.010
1q polysomy	17.5 (13.404–21.596)	0.056	–	
19p polysomy	17.0 (13.835–20.165)	0.009	1.930 (1.064–3.502)	0.031

*N/A* not available, *OR* odd ratio, *CI* confidence interval

**Fig. 1:**
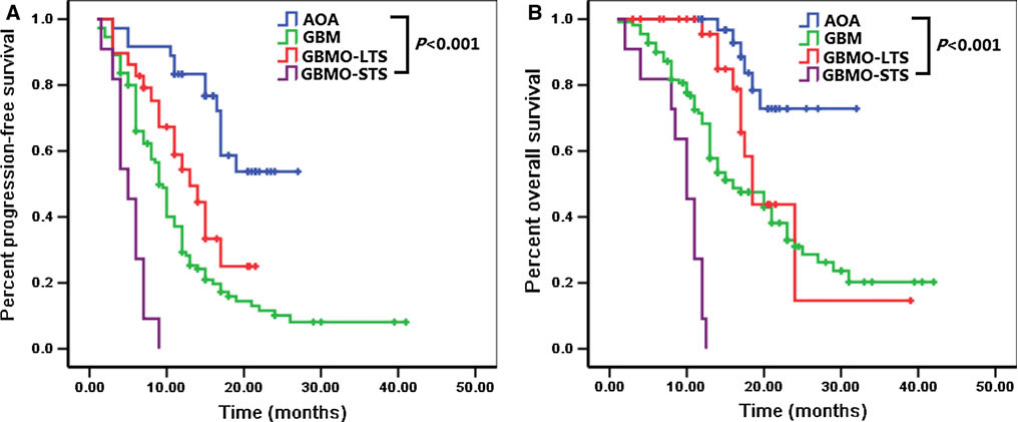
Kaplan–Meier plots for PFS and OS between AOA, GBMO-STS, GBMO-LTS and GBM were significantly different (*P* < 0.001 for PFS, *P* < 0.001 for OS, respectively)

The median PFS and OS of GBMO-STS were 5.0 [95 % CI 3.382–6.618] and 10.0 [95 % CI 7.977–12.023] months, respectively, which were significantly shorter than AOA, GBMO-LTS or GBM (*P* < 0.001 for PFS, *P* < 0.001 for OS, respectively) (Fig. 1 and Table S2).

**Fig. 2:**
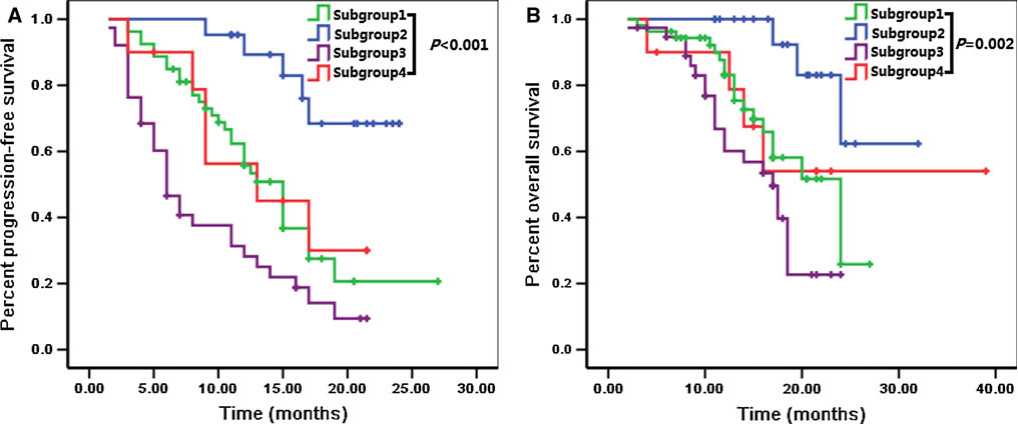
Subgroup 1, without co-deletion or polysomy; Subgroup 2, with co-deletion but without polysomy; Subgroup 3, with polysomy but without co-deletion; Subgroup 4, with polysomy and co-deletion. The survival time of subgroup 1 and 4 was significantly shorter compared with subgroup 2 (*P* = 0.001 for PFS, *P* = 0.015 for OS, respectively) but longer than subgroup 3 (*P* = 0.001 for PFS, *P* = 0.05 for OS, respectively). No significant difference of prognosis was observed between subgroup 1 and 4 (*P* = 0.803 for PFS, *P* = 0.868 for OS, respectively)

According to the status of chromosomes 1 and 19 (co-deletion yes or no, and polysomy yes or no), we could classified the 186 patients into four subgroups (subgroup 1, without co-deletion or polysomy; subgroup 2, with co-deletion but without polysomy; subgroup 3, with polysomy but without co-deletion; subgroup 4, with polysomy and co-deletion) which conferred different survival time. Patients in subgroup 2 exhibited the most favorable prognosis compared with subgroup 1, 3 and 4 (*P* < 0.001 for PFS, *P* = 0.002 for OS, respectively). Patients in subgroup 3 had the shortest survival time that the median PFS was 11.0 months and the OS was only 17.5 months. No significant difference of prognosis was observed between subgroup 1 and 4 (*P* = 0.803 for PFS, *P* = 0.868 for OS, respectively) (Fig. 2).

### Frequencies of 1p/19q co-deletion, IDH1 mutation and 1q, 19p polysomy in AOA, GBMO-STS, GBMO-LTS and GBM

Fluorescence in situ hybridization (FISH) for chromosome 1p and 19q was available in 121 cases. Among them, 28 (23.1 %) had 1p/19q co-deletion (including 14 in AOA, 0 in GBMO-STS, 11 in GBMO-LTS and 3 in GBM). The frequencies of 1p/19q co-deletion in AOA, GBMO-STS, GBMO-LTS and GBM were 40.0, 0.0 %, 37.9 and 6.5 %, respectively. The frequency of 1p/19q co-deletion in GBMO-STS was significantly lower than those in AOA or GBMO-LTS (*P* = 0.032 and *P* = 0.045, respectively).

DNA sequencing for IDH1 was available in 137 patients. Among them, 38 out of 137 (27.7 %) had IDH1 mutation (including 14 in AOA, 0 in GBMO-STS, 16 in GBMO-LTS and 8 in GBM). The frequencies of IDH1 mutation in AOA, GBMO-STS, GBMO-LTS and GBM were 42.4, 0.0, 55.2 and 12.3 %, respectively. The frequency of IDH1 mutation in GBMO-STS was significantly lower than those in AOA or GBMO-LTS (*P* = 0.034 and *P* = 0.005, respectively).

Chromosome polysomy status could be assessed in 123 patients. Of the 123 patients, 40 (32.5 %) had 1q ploysomy (including 8 in AOA, 7 in GBMO-STS, 13 in GBMO-LTS and 12 in GBM); 46 (37.4 %) had 19p ploysomy (including 9 in AOA, 8 in GBMO-STS, 16 in GBMO-LTS and 13 in GBM). 1q polysomy was slightly more frequent in GBMO-STS compared with AOA or GBM (*P* = 0.037 and *P* = 0.030, respectively). Furthermore, GBMO-STS exhibited higher 19p polysomy rate than AOA or GBM (*P* = 0.017 and *P* = 0.011, respectively) (Table 3).

**Table 3:** Genetic aberrations in AOA, GBMO-STS, GBMO-LTS, and GBM

Characteristic	Subgroup 1		*P* value	Subgroup 2		*P* value	Subgroup 3		*P* value
	GBMO-STS (*n* = 11)	AOA (*n* = 36)		GBM (*n* = 110)	GBMO-STS (*n* = 11)		GBMO-LTS (*n* = 29)	GBMO-STS (*n* = 11)	
1p/19q co-deletion									
Yes (%)	0 (0.0) (*n* = 11)	14 (40.0) (*n* = 35)	0.032*	3 (6.5) (*n* = 46)	0 (0.0) (*n* = 11)	1.0^#^	11 (37.9) (*n* = 29)	0 (0.0) (*n* = 11)	0.045*
IDH1 mutation									
Yes (%)	0 (0.0) (*n* = 10)	14 (42.4) (*n* = 33)	0.034*	8 (12.3) (*n* = 65)	0 (0.0) (*n* = 10)	0.533	16 (55.2) (*n* = 29)	0 (0.0) (*n* = 10)	0.005*
1q polysomy									
Yes (%)	7 (63.6) (*n* = 11)	8 (23.5) (*n* = 34)	0.037*	12 (24.5) (*n* = 49)	7 (63.6) (*n* = 11)	0.030*	13 (44.8) (*n* = 29)	7 (63.6) (*n* = 11)	0.288
19p polysomy									
Yes (%)	8 (72.7) (*n* = 11)	9 (26.5) (*n* = 34)	0.017*	13 (26.5) (*n* = 49)	8 (72.7) (*n* = 11)	0.011*	16 (55.2) (*n* = 29)	8 (72.7) (*n* = 11)	0.515
MGMT promoter									
Methylated (%)	2 (18.2) (*n* = 11)	16 (44.4) (*n* = 36)	0.225	16 (34.8) (*n* = 46)	2 (18.2) (*n* = 11)	0.482	10 (34.5) (*n* = 29)	2 (18.2) (*n* = 11)	0.536
PTEN									
Negative expression (%)	1 (12.5) (*n* = 8)	6 (20.7) (*n* = 29)	1.0^#^	7 (17.1) (*n* = 41)	1 (12.5) (*n* = 8)	1.0	6 (20.7) (*n* = 29)	1 (12.5) (*n* = 8)	1.0^#^
P53									
Negative expression (%)	2 (25.0) (*n* = 8)	7 (24.1) (*n* = 29)	1.0^#^	11 (26.8) (*n* = 41)	2 (25.0) (*n* = 8)	1.0	6 (20.7) (*n* = 29)	2 (25.0) (*n* = 8)	1.0^#^
Ki-67									
Negative expression (%)	1 (12.5) (*n* = 8)	13 (44.8) (*n* = 29)	0.123^#^	14 (34.1) (*n* = 41)	1 (12.5) (*n* = 8)	0.426	9 (31.0) (*n* = 29)	1 (12.5) (*n* = 8)	0.404^#^
EGFR									
Negative expression (%)	1 (12.5) (*n* = 8)	7 (24.1) (*n* = 29)	0.655^#^	4 (9.8) (*n* = 41)	1 (12.5) (*n* = 8)	1.0^#^	5 (17.2) (*n* = 29)	1 (12.5) (*n* = 8)	1.0^#^
VEGF									
Negative expression (%)	1 (12.5) (*n* = 8)	2 (6.9) (*n* = 29)	0.530^#^	2 (4.9) (*n* = 41)	1 (12.5) (*n* = 8)	0.421^#^	3 (10.3) (*n* = 29)	1 (12.5) (*n* = 8)	1.0^#^

*MGMT* O6-methylguanine-DNA-methyltransferase, *PTEN* phosphatase and tensin homolog, *EGFR* epidermal growth factor receptor, *VEGF* vascular endothelial growth factor

^#^ Fisher’s exact test

## Discussion

Anaplastic oligoastrocytoma (AOA) with necrosis, formerly categorized in WHO grade III, now is regarded as GBMO (WHO grade IV) which is a heterogeneous group with considerable survival variant. Several reports dedicated to the survival analysis about GBM versus GBMO showed that no favorable prognostic value of an oligodendroglial component was found [4–6], although some other studies indicated a longer survival for GBMO [1–3, 10] (Table 4). In the present study, we found a subtype of GBMO harbored shorter survival time compared with GBM. This is, so far as we know, the first series of GBMO short-term survivors reported to date. We provide a clinical characterization and report on molecular analyses of the 11 patients who have a survival time ≤12 months in order to reveal the reasons for the dismal prognosis.

**Table 4:** The outcomes of selected series of GBMO and GBM

Author	Patient group	Number of patients	Treatment	Median OS (months)	*P* value
Miller et al. [2]	AOA with necrosis	71	N/A	22.8	
	GBM	581	N/A	9.8	<0.0001*
Vordermark et al. [1]	GBMO	10	Post-OPT RT in 90 % + ACNU and VM26 in 80 %	26.0	N/A
Kanno et al. [3]	GBMO	17	Post-OPT RT + ACNU + TMZ	≈40.0^a^	
	GBM	52	Post-OPT RT + ACNU + TMZ	≈18.0^a^	0.068
Jiang et al. [10]	GBMO	40	Post-OPT RT in 85 %; RT + Chemo in 62 %	19.0	
	GBM	179	Post-OPT RT in 87 %; RT + Chemo in 65 %	13.2	0.022*
Pinto et al. [4]	GBMO	24	Post-OPT RT + Chemo	14.9	
	GBM	64	Post-OPT RT + Chemo	13.5	0.566
Hegi et al. [5]	GBMO	52	Post-OPT RT + Concomitant TMZ + Ajuvand TMZ	N/A	
	GBM	287	Post-OPT RT + Concomitant TMZ + Ajuvand TMZ	N/A	0.48
Nakamura et al. [6]	GBMO	19	Post-OPT RT in 100 % + ACNU/TMZ in 89.5 %	14.0	N/A
Present study	GBMO-STS	11	Post-OPT RT in 100 % + ACNU in 100 % + TMZ in 63.6 %	10.0	
	GBMO-LTS	29	Post-OPT RT in 96.6 % + ACNU in 89.7 % + TMZ in 72.4 %	18.5	
	GBM	110	Post-OPT RT in 92.7 % + ACNU in 96.4 % + TMZ in 53.6 %	16.0	<0.001*

^a^Estimated value from graph

*OPT* operation, *RT* radiation therapy, *Chemo* chemotherapy, *ACNU* nimustine, *TMZ* temozolomide, *N/A* not available

### No significant difference in clinical characterization was found between AOA, GBMO-STS, GBMO-LTS and GBM

Considering variable prognostic factors such as the age of the patients, extent of resection, postoperative radiotherapy or chemotherapy, Karnofsky performance status (KPS) influenced the patients’ survival, we recruited all the clinical factors in the present study. We found that except for the age at diagnosis of patients with GBM was older than those in GBMO-STS (*P* = 0.048), no other significant difference was observed between the four subgroups (AOA, GBMO-STS, GBMO-LTS and GBM) (Table 1). But the prognosis of patients with GBM was, unexpectedly, better than GBMO-STS. It suggested the prognostic value of age was covered after the adjustment for some potential prognostic factors, such as 1p/19q co-deletion, polysomy for 1q and 19p, in these high-grade gliomas.

### High-frequency of 1p/19q co-deletion and IDH1 mutation result in the survival advantage of AOA and GBMO-LTS

In our cohort, we found that the GBMO-STS exhibited lower frequency of 1p/19q co-deletion than AOA or GBMO-LTS but resembled it with GBM. This finding was similar with Jiang’s report, which maintained there was no significant difference of 1p/19q co-deletion rate between GBMO and GBM [10]. 1p/19q co-deletion is an established genetic marker for prognostication about glioma patients’ survival and chemosensitivity [11, 12]. In 1998, Cairncross and colleagues reported that loss of 1p (and 1p/19q co-deletion) predicts a better response to procarbazine-lomustine-vincristine chemotherapy and a longer survival in patients with AO [13]. These findings have been reproduced in many subsequent studies, including prospective and randomized phase III trials [14, 15]. Moreover, oligodendroglial tumors with loss of 1p/19q showed a response to treatment with the alkylating drug TMZ and radiotherapy, indicating its predictive value for a broader spectrum of therapeutic regimens [16–18]. These results indicated that patients with AOA or GBMO-LTS had a significantly longer survival time than GBMO-STS might be linked to the higher incidence of 1p/19q co-deletion.

As elaborated by experts, IDH1 mutation was associated with a better outcome in patients with low-grade diffuse gliomas, AA, GBM and had been shown to be a powerful independent prognostic factor for prolonged survival [19, 20]. IDH1/2 genes encode for the cytosolic and mitochondrial nicotinamide adenine dinucleotide phosphate (NADPH)-dependent isocitrate dehydrogenase enzymes which play an vital role in the citric acid cycle. Wild-type IDH1/2 isozymes catalyze the oxidative carboxylation of isocitrate to a-ketoglutarate and reduce NADP+ to NADPH during this process [21, 22]. Both the a-ketoglutarate and the released NADPH are known cell defenders against oxidative damage. Mutated IDH gene decreases the ability of the IDH enzyme to catalyze the conversion of isocitrate to a-ketoglutarate and leads to a decreased quantity of a-ketoglutarate and NADPH, making the cell more susceptible to oxidative stress [23]. In the present study, patients of GBMO-STS showed lower IDH1 mutation rate compared with AOA or GBMO-LTS, which suggested that the survival of GBMO-STS would be shorter than AOA or GBMO-LTS. However, the incidence of IDH1 mutation, in our cohort, was a little lower in comparison to the reports from Europe or America, but it was similar with Jiang’s report which displayed the frequency of IDH1/2 mutation in Chinese AOA was 45.8 %. Ethnic differences might partly explain it [24–26]. Inevitably, the phenomenon might, to some extent, due to our imperfect experiment method. Of interest, there was no significant difference of IDH1 mutation rate between GBMO-STS and GBM. It documented that the incidence of IDH1 mutation could not be a parameter which resulted in the difference of survival time between the two groups.

### Presence of 1q, 19p polysomy contributed to the dismal prognosis of GBMO-STS

Univariate analysis revealed that polysomy for 1q and 19p were associated with the dismal prognosis of patients with high-grade gliomas. These findings were in consistent with many previous reports. Snuderl et al. [27] reported that polysomy for chromosomes 1 and 19 predicted earlier recurrence in anaplastic oligodendrogliomas with concurrent 1p/19q loss. Wiens et al. [28] documented that combined polysomy was associated with higher histological tumor grade and conferred poor survival likelihood. They concluded polysomy of 1q and/or 19p was a relatively frequent occurrence in oligodendrogliomas and usually conferred an unfavorable outcome. In the present study, intergroup comparison showed GBMO-STS harbored higher frequency of 1q, 19p polysomy than AOA or GBM. It might partly interpret the phenomenon that GBMO-STS with a median OS of merely 10.0 months which was significantly shorter than AOA or even GBM. Another possible explanation for the dismal prognosis of GBMO was that these tumors were in fact small cell GBM which exhibited shorter survival time than GBM and could mimic GBMO. Considering this issue, a pathology re-evaluation was performed. Though the pathology consultation result revealed these specimens were GBMO, the EGFR amplification information was the best means to making the distinction between small cell GBM and GBMO. Because of the limited experimental resource, the EGFR amplification was not available in our laboratory. Finally, higher incidence of polysomy for 1q and 19p might be a potential parameter which contributed to the survival time ≤12 months of GBMO-STS.

From the above, experts, all over the world, in regard of the prognostic value of an oligodendrogial component in glioblastomas couldn’t arrive at a consensus (Table 4). Based on the data displayed in the study, we speculate that GBMO, a heterogeneous group with considerable survival variant, directly being regarded as “glioblastomas” with relatively favorable outcome remains a subject needs further inquiry. Because there is a subtype GBMO with concurrent polysomy for chromosomes 1 and 19 exhibits shorter survival than GBM. When encountered with such subtype of patients, perhaps taking the polysomy for chromosomes 1 and 19 into account would be more reasonable in guiding the individual therapy in clinic care. Another important issue is that FISH seems to be more suitable for assessing loss of 1p and 19q compared with polymerase chain reaction (PCR) with regard to clinical significance of polysomy. Because, compared with PCR-based loss of heterozygosity (LOH) assays, additional polysomy information can be gleaned from the FISH analysis.

### Study limitation

The small sample of GBMO-STS which would weaken the conviction of this study to some extent. So we will enlarge our sample for the further inquiry in the future. It was also a limitation that the EGFR amplification information was absent in distinguishing GBMO from small cell GBM.

## Conclusions

Patients with GBMO concurrent with polysomy for chromosomes 1 and 19 always confers an unfavorable prognosis which needs our extra attention in clinic.

## Electronic supplementary material

Below is the link to the electronic supplementary material.

Supplementary material 1 (TIFF 197 kb)

Supplementary material 2 (DOCX 22 kb)

Supplementary material 3 (DOCX 15 kb)
