# Supplementary figures and images for: Short-term survivors in glioblastomas with oligodendroglioma component: a clinical study of 186 Chinese patients from a single institution
Source: J Neurooncol. 2013 Nov 22;116(2):395–404. doi: 10.1007/s11060-013-1311-3 (PMC3890040; doi:10.1007/s11060-013-1311-3)

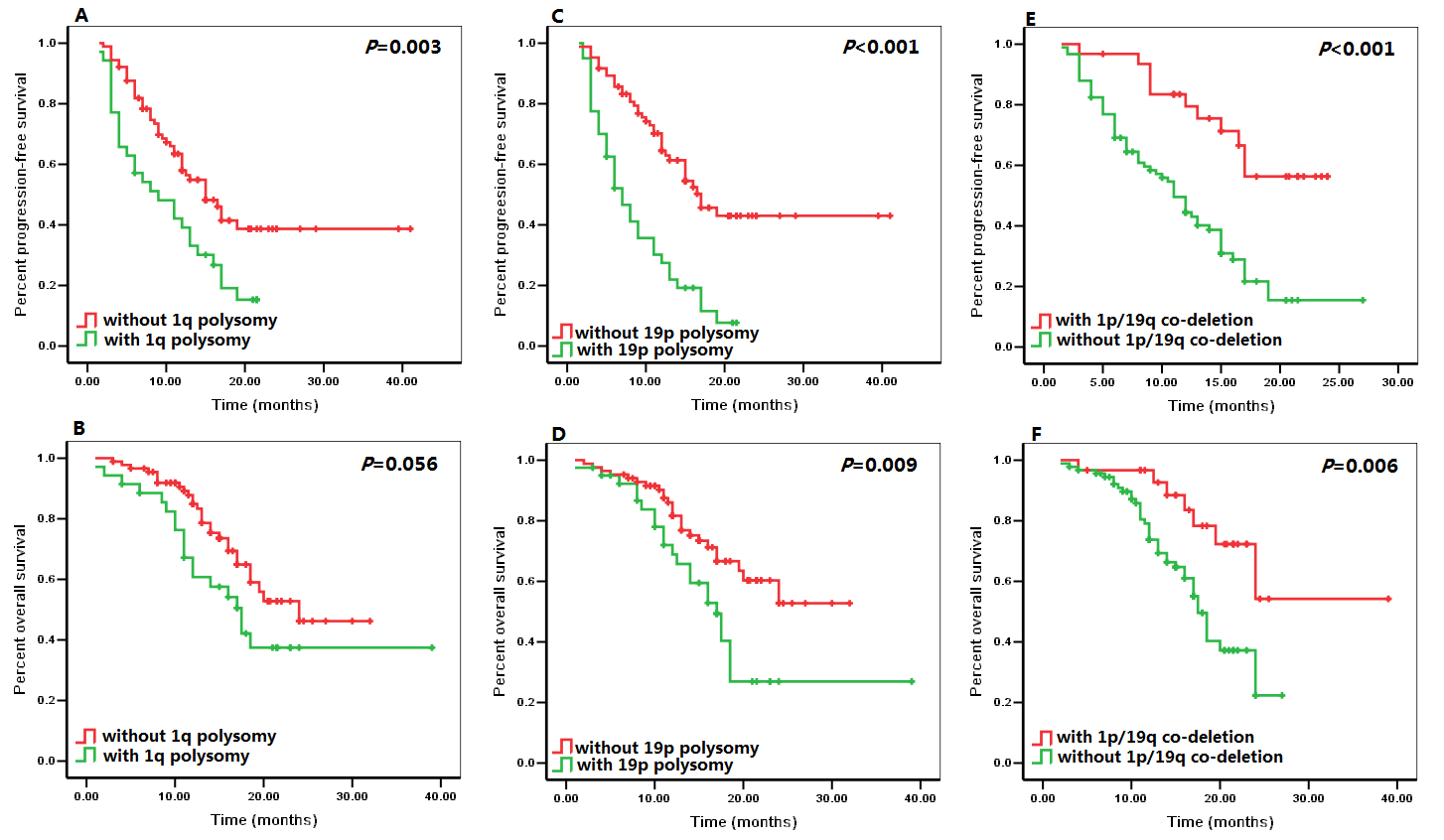

Supplement: Supplementary file 1 — Supplementary material 1 (TIFF 197 kb) [file 11060_2013_1311_MOESM1_ESM.tif]
