# Supplementary material for: Short-term survivors in glioblastomas with oligodendroglioma component: a clinical study of 186 Chinese patients from a single institution
Source: J Neurooncol. 2013 Nov 22;116(2):395–404. doi: 10.1007/s11060-013-1311-3 (PMC3890040; doi:10.1007/s11060-013-1311-3)
Supplement: Supplementary file 2 — Supplementary material 2 (DOCX 22 kb) [file 11060_2013_1311_MOESM2_ESM.docx]

Table S1**.** Antibodies for immunohistochemical staining

| **Antibodies** | **Company** | **Dilution** | **Ag retrieval technique** |
| --- | --- | --- | --- |
| EGFR | Invitrogen, CA, USA | 1:100 | Pepsin 10 min at 37°C |
| VEGF | Zeta, CA, USA | 1:150 | Microwave 12 min |
| P53 | Invitrogen, CA, USA | 1:50 | Microwave 12 min |
| Ki-67 | Invitrogen, CA, USA | 1:150 | Microwave 12 min |
| PTEN | Lab Vision, CA, USA | 1:150 | Microwave 12 min |

Abbreviations: PTEN, Phosphatase and tensin homolog; EGFR Epidermal growth factor receptor; VEGF, Vascular endothelial growth factor.
