# Supplementary material for: Short-term survivors in glioblastomas with oligodendroglioma component: a clinical study of 186 Chinese patients from a single institution
Source: J Neurooncol. 2013 Nov 22;116(2):395–404. doi: 10.1007/s11060-013-1311-3 (PMC3890040; doi:10.1007/s11060-013-1311-3)
Supplement: Supplementary file 3 — Supplementary material 3 (DOCX 15 kb) [file 11060_2013_1311_MOESM3_ESM.docx]

Table S2. The clinical outcomes AOA, GBMO-STS, GBMO-LTS and GBM

| **Variable** | **Median PFS (95% CI)** | **Median OS (95% CI)** | ***P*-value** |
| --- | --- | --- | --- |
| AOA | N/A | N/A |  |
| GBMO-STS | 5.0 (3.382-6.618) | 10.0 (7.977-12.023) |  |
| GBMO-LTS | 13.0 (8.848-17.152) | 18.5 (16.767-20.233) |  |
| GBM | 9.0 (8.136-9.864) | 16.0 (11.290-20.710) | <0.001 |
| Abbreviations: N/A, not available; PFS, progression-free survival; OS, overall survival; CI=confidence interval. | | | |
